# HIV-1 Tat Protein Promotes Neuroendocrine Dysfunction Concurrent with the Potentiation of Oxycodone’s Psychomotor Effects in Female Mice

**DOI:** 10.3390/v13050813

**Published:** 2021-04-30

**Authors:** Mohammed F. Salahuddin, Fakhri Mahdi, Suresh P. Sulochana, Jason J. Paris

**Affiliations:** 1 Department of BioMolecular Sciences, University of Mississippi School of Pharmacy, Oxford, MS 38677-1848, USA; smohamme@go.olemiss.edu (M.F.S.); fmahdi@olemiss.edu (F.M.); 2 Department of Pharmaceutics and Drug Delivery, University of Mississippi School of Pharmacy, Oxford, MS 38677-1848, USA; spsuloch@olemiss.edu; 3 Research Institute of Pharmaceutical Sciences, University of Mississippi School of Pharmacy, Oxford, MS 38677-1848, USA

**Keywords:** antalarmin, hypothalamic-pituitary-adrenal axis, ovariectomy, opioids, mifepristone (RU-486), trans-activator of transcription

## Abstract

Human immunodeficiency virus (HIV) is associated with neuroendocrine dysfunction which may contribute to co-morbid stress-sensitive disorders. The hypothalamic-pituitary-adrenal (HPA) or -gonadal (HPG) axes are perturbed in up to 50% of HIV patients. The mechanisms are not known, but we have found the HIV-1 trans-activator of transcription (Tat) protein to recapitulate the clinical phenotype in male mice. We hypothesized that HPA and/or HPG dysregulation contributes to Tat-mediated interactions with oxycodone, an opioid often prescribed to HIV patients, in females. Female mice that conditionally-expressed the Tat_1–86_ protein [Tat(+) mice] or their counterparts that did not [Tat(−) control mice] were exposed to forced swim stress (or not) and behaviorally-assessed for motor and anxiety-like behavior. Some mice had glucocorticoid receptors (GR) or corticotropin-releasing factor receptors (CRF-R) pharmacologically inhibited. Some mice were ovariectomized (OVX). As seen previously in males, Tat elevated basal corticosterone levels and potentiated oxycodone’s psychomotor activity in females. Unlike males, females did not demonstrate adrenal insufficiency and oxycodone potentiation was not regulated by GRs or CRF-Rs. Rather OVX attenuated Tat/oxycodone interactions. Either Tat or oxycodone increased anxiety-like behavior and their combination increased hypothalamic allopregnanolone. OVX increased basal hypothalamic allopregnanolone and obviated Tat or oxycodone-mediated fluctuations. Together, these data provide further evidence for Tat-mediated dysregulation of the HPA axis and reveal the importance of HPG axis regulation in females. HPA/HPG disruption may contribute vulnerability to affective and substance use disorders.

## 1. Introduction

Human immunodeficiency virus type 1 (HIV-1) infection remains a significant public health concern with ~1.1 million people living with HIV in the U.S. The widespread use of combined antiretroviral therapeutics (cART) has largely increased life expectancy among HIV-infected patients; however, the inability of cART to target latent central nervous system (CNS) reservoirs including microglia and astrocytes [1], likely contributes to the persistence of central viremia and neurological symptomatology [2,3]. Approximately 50% of the HIV^+^ population contend with a constellation of neurological symptoms (collectively referred to as “neuroHIV”) that include affective disorders (i.e., increase generalized anxiety and major depression), psychomotor deficits and cognitive impairments [4,5,6]. CNS complications also include neuropathic pain resulting in 8–52% of HIV-1 patients being prescribed opioids [7,8,9,10,11,12] which may interact with HIV-1 proteins, complicating outcomes. Thus, despite progress attenuating systemic viremia, the central compartment remains an important target for therapeutic advancement.

While the mechanisms that underlie neuroHIV remain the subject of intense investigation, neurotoxic HIV-1 proteins can persist within in the CNS where cART is poorly retained (indicated by the presence of viral proteins in cerebrospinal fluid [13,14]). In particular, the HIV-1 trans-activator of transcription (Tat) is critical for efficient viral transcription [15] but can also exert neural damage via multiple mechanisms including the promotion of neuroinflammation [16,17], direct excitotoxic injury to neurons [18,19,20] and the promotion of mitochondrial dysregulation and mitotoxicity [18,21,22,23]. In particular, disruption of mitochondrial activity in the CNS may contribute to neuroendocrine dysfunction observed in HIV^+^ patients given that mitochondria are the rate-limiting organelle necessary for steroidogenesis.

While under-investigated, up to half of HIV-1 patients suffer from neuroendocrine dysfunction that is secondary in etiology (i.e., occurring at the level of the CNS, not the primary steroid-producing glands). HPA dysfunction (elevated basal cortisol levels and paradoxical adrenal insufficiency in response to a stressor) is reported in 14–46% of HIV-1 patient samples [24,25,26,27,28]. Hypogonadism (reduced testosterone production in men and dysregulated estradiol/progestogen levels in women) is reported in 10–50% of HIV-1 samples [29,30,31,32,33,34,35,36]. Perturbation of the hypothalamic-pituitary-adrenal (HPA) or -gonadal (HPG) neuroendocrine axes would be expected to contribute to neuroHIV symptomatology and/or vulnerability. The HPA axis plays a critical role in the recovery from a variety of stressors including immune, psychological or drug of abuse challenges [37,38,39,40]. Prolonged HPA/HPG dysfunction may promote vulnerability to stress-sensitive neuropsychiatric disorders and addiction [41]. Using a transgenic mouse model, we have found conditional expression of HIV-1 Tat in males to disrupt CNS steroidogenesis [23], to elevate basal production of the circulating stress hormone, corticosterone, and to produce adrenal insufficiency in response to a physical or pharmacological HPA challenge [42]. Importantly, HPA dysfunction was observed concurrent with an increased psychomotor response to the clinical opioid, oxycodone [42]. However, the interactions with endocrine function in females may be more complex and is less well-understood. We have observed elevated circulating corticosterone and hypothalamic corticotropin releasing factor (CRF) in Tat- or oxycodone-exposed female mice to be indicative of HPA activation [43]. However, it was not known whether females expressed the adrenally insufficient profile observed in males, what central targets may contribute to these effects, or how their greater estrane/pregnane gonadal steroid capacity would influence outcomes. As such, these potential sex differences in Tat-mediated HPA dysfunction were sought in the present work.

We hypothesized that conditional expression of HIV-1 Tat in female mice would produce HPA dysregulation, potentiate oxycodone’s psychomotor effects and promote anxiety-like behavior. We anticipated these effects to be partly mediated by negative regulators of the HPA axis, glucocorticoid receptors (GR) and/or CRF receptors (CRF-R), as was previously observed in males [42]. We further anticipated that peripherally- or centrally-produced steroids would modify the HPA response.

## 2. Materials and Methods

The Institutional Animal Care and Use Committee (IACUC) at University of Mississippi preapproved the procedures and protocols for the present study (#18-004 & 21-005; approved on 25 October 2017 & 28 September 2020 respectively). All the experiments were performed in accordance with guidelines defined by the National Institute of Health (NIH; Publication No. 85-23).

### 2.1. Subjects & Housing

Female, adult, HIV-1 Tat-transgenic mice (*n* = 313) were bred in the vivarium at the University of Mississippi (Oxford, MS, USA). Mice were housed (2–5/cage) and maintained in a humidity and temperature-controlled environment on a 12:12 h reverse light:dark cycle (lights off at 09:00 h) with *ad libitum* access to food and water. Briefly, mice expressed the Tat_1–86_ protein via conditional, GFAP-relegated expression under regulation of a reverse tetracycline-controlled transactivating (rtTA) transcription factor. We and others have previously confirmed mRNA expression of *tat* in the brain and spinal cord of these mice [44,45,46] and include confirmation in the present tissues via quantitative real-time PCR (see Supplemental Data, Figure S1). Tat(−) control mice lacked the *tat* transgene but did express the rtTA transcription factor [47]. While rtTA gene expression is notably ‘leaky’ [48], Tat(−) controls lack the *tat* transgene making them an optimal comparison group. Moreover, we have previously demonstrated that young-adult Tat(+) mice do not demonstrate *tat*-mediated behavioral interactions with drugs of abuse until doxycycline-induction [23] and include a saline-administered, un-induced control group herein (see Supplemental Data, Figure S2).

### 2.2. Surgical Manipulation

A subset of mice (*n* = 32) underwent bilateral ovariectomy under isoflurane anesthesia (2.5–4.0%) per previous methods [49]. Post-surgery, mice were transferred to a clean cage with food, water, and access to acetaminophen (2 mg/mL) for a period of 96 h. Weight, neurological status, surgical site, and food consumption were monitored daily [50]. Two mice failed to recover and were thus excluded from the study. Mice were allowed 7 days of recovery to allow for endogenous hormone washout prior to additional pharmacological manipulations.

### 2.3. Chemicals

**Figure 1:**
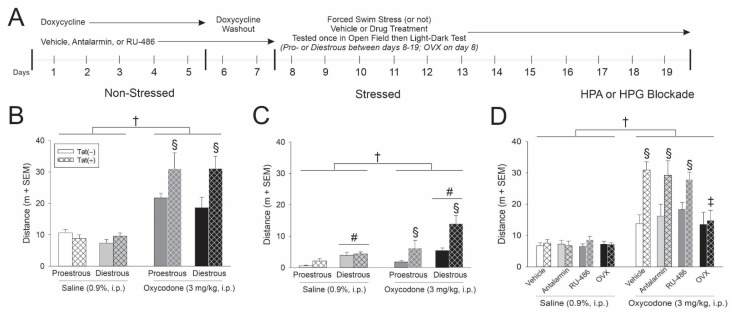
(**A**) HIV-1 Tat-transgenic, female mice [hatched bars; Tat(+)] or control counterparts [open bars; Tat(−) controls] had transgene expression induced via doxycycline (30 mg/kg, i.p., once daily for 5 days with 2 days for washout; *n* = 8–10/group). Some mice were pretreated with either vehicle. antalarmin, RU-486 or were ovariectomized (OVX). On the day of testing, proestrous, diestrous, or OVX mice were exposed to a forced swim stress (or not) and administered saline or oxycodone 15 min prior to assessment in an open field and light-dark transition test. (**B**) Distance (m) travelled in an open field among (**B**) non-stressed mice, (**C**) stressed mice and (**D**) mice that were pretreated with antalarmin or RU-486 or were OVX. † indicates a main effect for oxycodone-administered mice to differ from saline-administered controls. § indicates an interaction wherein oxycodone-administered Tat(+) mice differ from respective Tat(−) controls and saline-administered controls. # indicates a main effect of estrous cycle wherein diestrous mice differ from proestrous mice. ‡ indicates an interaction wherein oxycodone-administered Tat(+) OVX mice differ from all other oxycodone- administered Tat(+) groups, *p* < 0.05.

Doxycycline hyclate (Cayman Chemical, Ann Arbor, MI, USA) was made fresh daily to a dose of 30 mg/kg in sterile saline (0.9% *w*/*v*) and injected via intraperitoneal (i.p.) route once daily for 5 days. Oxycodone HCl (Sigma-Aldrich, St. Louis, MO, USA) was dissolved to a concentration of 3.0 mg/kg in sterile saline and injected i.p. 15 min prior to behavioral testing (see Figure 1A). The present dose of oxycodone (3.0 mg/kg) was identified as the minimum effective dose to diverge psychomotor responding between Tat(−) and Tat(+) mice [43]. To pharmacologically block CRF-receptors, mice were administered antalarmin (Cayman Chemical) dissolved in 30% Kolliphor (70% sterile saline) to a dose of 20.0 mg/kg (i.p) administered once daily for 7 days [42,51,52]. To block glucocorticoid receptors, mice were administered RU-486 (a.k.a. mifepristone; Cayman Chemical) dissolved in 30% Kolliphor, 1% DMSO and Tween 20 (3 drops) to a dose of 20 mg/kg (i.p.) administered once daily for 8 days [42,53,54].

### 2.4. Determination of Estrous Cycle Phase

Estrous cycle was determined via vaginal lavage conducted daily at 09:00 h. Cytology was assessed to determine the estrous cycle phase as modified from previous descriptions [43,55]. Mice were tested when in the proestrous or diestrous phase of their estrous cycle in order to control for hormonal variations that might influence behavioral responses. In the current study, no significant differences were observed between mice on diestrus I (a.k.a. metestrus) or diestrus II; as such, these groups were combined.

### 2.5. Behavioral Assays

#### 2.5.1. Forced Swim Stress

To activate the HPA stress axis, mice were exposed to a forced swim stimulus as previously described [42]. Briefly, mice were placed in a container filled with room temperature water (approximately 22 °C) and permitted to swim for 15 min. At the end of the swim stressor, mice were dried and returned to their home cages. Following swim stress mice were assessed in a behavioral battery comprised of an open field and a light-dark transition test (described below).

#### 2.5.2. Open Field Test

The open field test was used to assess spontaneous motor behavior as previously described [42,43]. Briefly, mice were placed gently in a corner of a square-shaped open field box (40 × 40 × 35 cm^3^; Stoelting Co., Wood Dale, IL, USA) with a brightly-lit center (inner 20 × 20 cm^2^) and allowed to behave for 5 min. Dependent measures included overall distance travelled (in meters) and mean velocity (m/s) as indices of spontaneous motor behavior.

#### 2.5.3. Light-Dark Transition Test

Following testing in the open field, mice were assessed for anxiety-like behavior in the light-dark transition test. Given the psychostimulatory effects of opioids, the light-dark test was preferred given that anxiety-like indices are less confounded by manipulations that affect motor behavior [56]. The light-dark apparatus consisted of two compartments, one brightly-lit (20 × 20 × 35 cm^3^) and the other dark and enclosed (20 × 20 × 35 cm^3^; Stoelting Co., Wood Dale, IL, USA). Briefly, mice were placed in a corner of the brightly-lit side of the apparatus and permitted to behave for 5 min. The latency to enter the dark chamber and the total time spent in the light zone were considered indices of anti-anxiety-like behavior.

### 2.6. Biochemical Assays

#### 2.6.1. Tissue Collection

Following behavior testing, mice underwent cervical dislocation followed by rapid decapitation. Whole brains and trunk blood were collected. Brains were immediately flash-frozen on dry ice. Blood was collected in a chilled aliquot tube and serum was separated via centrifugation at 13,500× *g*. Tissues and serum were stored at −80 °C. Hypothalamus was later grossly dissected on ice as previously described [43] and frozen at −80 °C until assay.

#### 2.6.2. Enzyme-Linked Immunosorbant Assay (ELISA)

Serum steroid extraction was achieved via ether-snap freezing. Briefly, 1 mL of anhydrous ethyl ether (ice-cold) was added to serum samples which were vortexed and snap frozen using a dry ice/acetone mixture as described previously [43]. Ether supernatant was collected and evaporated to dryness overnight in a chemical fume hood. Crystalline steroids were reconstituted 5× (for estradiol), 25× (for progesterone) or 50× (for corticosterone) their initial volume in assay extraction buffer (Neogen Life Sciences, Lexington, KY, USA).

Circulating levels of estradiol, progesterone and corticosterone were measured via ELISA per manufacturer-suggested methods (#402110, Estradiol; #402310, Progesterone; #402810, Corticosterone; Neogen Life Sciences) and as previously described [42,43]. All assays were read at an absorbance of 650 nm using a CLARIOstar microplate reader (BMG Labtech Inc., Cary, NC, USA). Respective intra-assay variances were: Estradiol: 9%, Progesterone: 8.3% and Corticosterone: 6.1%; inter-assay variances were: Estradiol: 26.7%, Progesterone: 29.1% and Corticosterone: 21.9%.

#### 2.6.3. Ultra-Performance Liquid Chromatography (UPLC)-Mass Spectrometry (MS)

For UPLC-MS/MS, charcoal-stripped tissue (brain tissue derived from Tat-tg mice) was utilized to prepare both the calibration curve and quality control samples for analysis. A simple protein precipitation method was used for steroid extraction. Samples were homogenized (100 μL of PBS pH 7.4) and precipitated with 100 µL of acetonitrile followed by vortexing (2 min) and centrifugation (10 min at 14,000 rpm). After centrifugation, the supernatant solution was mixed with 50 μL of derivatizing solution (20 mg/mL of 2-hydrazinopyridine solution prepared in 0.5% trifluoroacetic acid ethanol solution) and incubated at 60 °C for 1 h. Following incubation, 20 μL of the internal standard solution (1 μg/mL) was added and vortex mixed. For sample analysis, aliquots of 2 μL were injected onto the UPLC-MS/MS instrument.

### 2.7. Procedure

All manipulations (apart from genotype and cycle phase) were randomly assigned. HIV-1 Tat was conditionally induced in female adult transgenic mice via doxycycline administration for 5 days (i.p.). Given that the anti-inflammatory effects of doxycycline could potentially mask some Tat-mediated effects, two days of doxycycline washout were included prior to testing [57]. Estrous cycle was determined daily, and mice were assessed when in the proestrous or diestrous phases. Given that effects of Tat are found to be stable for at least 3 weeks [45,58], mice were behaviorally assessed no more than 14 days from Tat induction (Figure 1A,B). Some mice underwent forced swim stress for 15 min (stressed paradigm) or not (non-stressed paradigm). Some mice were pretreated with vehicle, the CRF-R antagonist, antalarmin, or the GR antagonist, RU-486. Some mice were ovariectomized to remove the primary source of gonadal hormones and administered a daily vehicle injection (to account for potential injection stress). All mice received either acute oxycodone or vehicle saline 15 min prior to assessment in the open field and light-dark transition tests. Notably, mice that received vehicle, antalarmin, or RU-486 were administered a final dose 30 min prior to behavioral testing. To assess the restoration of the HPA axis response, all mice were sacrificed approximately 2 h after the initiation of behavior testing (a time when circulating stress steroids are resolving) as opposed to prior observations that were conducted at 30 min post-testing (a time of peak stress steroid response [42]).

### 2.8. Statistical Analyses

Biochemical endpoints (i.e., circulating and central steroid measures) were initially utilized as covariates in multivariate analyses of variance (MANOVA) but were not found to explain significant variance in primary dependent measures. Thus, behavioral and biochemical dependent measures were assessed via separate two to three-way analyses of variance (ANOVA) with estrous cycle phase (proestrous or diestrous), pretreatment (vehicle, oxycodone, antalarmin, RU-486, or OVX), and/or genotype [Tat(−) or Tat(+)] as between-subject factors. Group differences following main effects were determined via Fisher’s Protected Least Significant Difference *post hoc* tests. Significant interactions were delineated via simple main effects and main effect contrasts with α controlled for family-wise error. All analyses were considered significant when *p* < 0.05.

## 3. Results

### 3.1. HIV-1 Tat and Oxycodone-Mediated Psychostimulation Is Moderated by Stress and Estrous Cycle

In order to assess combined HIV-1 Tat interactions with oxycodone, Tat was induced via systemic administration of doxycycline for 5 days (with two days of washout; Figure 1A). Estrous cycle was assessed daily over the next 12 days and mice were behaviorally tested when in the proestrus or diestrus phase of their estrous cycle (whichever came first). A 15 min forced swim was used to activate the HPA axis in the stressed paradigm (or not in the non-stressed paradigm). On the day of testing, all mice received saline or oxycodone (3 mg/kg, i.p.) 15 min prior to behavioral assessment (Figure 1A).

In the non-stressed paradigm, oxycodone significantly increased the distance [*F*(1,70) = 69.07, *p* < 0.05] (see †, Figure 1B) and velocity [*F*(1,70) = 69.42, *p* < 0.05] (see †, Table 1) travelled by the mice in an open field test compared to saline administered controls. There was an interaction wherein oxycodone-administered Tat(+) mice travelled a significantly greater distance (*p* < 0.0001; see §, Figure 1B) and speed (*p* < 0.0001–0.0002; see §, Table 1) than any other group, irrespective of estrous cycle phase. Oxycodone-administered Tat(−) controls also travelled a greater distance than their saline-administered counterparts (*p* = 0.0001–0.0002). When anxiety-like behavior was assessed in a light-dark transition test, estrous cycle, oxycodone, and Tat exposure interacted to influence the time spent in light zone [*F*(1,67) = 5.34, *p* < 0.05]. Diestrous Tat(+) mice administered saline demonstrated the least anxiety-like behavior, spending significantly more time in the light zone than any other group with the exception of proestrous Tat(+) mice administered saline (*p* = 0.0003–0.0478; Table 1). Diestrous Tat(+) mice administered oxycodone demonstrated the most anxiety-like behavior on this test, significantly differing from proestrous, Tat(+) controls (*p* = 0.0329; Table 1). As expected, oxycodone also influenced motor behavior in the light-dark test, significantly increasing the number of chamber transitions [*F*(1,69) = 16.10, *p* < 0.05] (see †, Table 1); whereas Tat(+) mice made significantly fewer transitions than Tat(−) controls [*F*(1,69) = 4.10, *p* < 0.05] (see *, Table 1). Estrous cycle also influenced motor/exploratory behavior in this test, with diestrous mice rearing more than proestrous mice [*F*(1,69) = 6.60, *p* < 0.05] (see #, Table 1).

In the stressed paradigm, motor activity was notably reduced compared to the non-stressed paradigm; however, the influence of estrous cycle phase became apparent after forced swim stress. As anticipated, oxycodone-administered mice travelled a significantly greater distance [*F*(1,71) = 16.51, *p* < 0.05] (see †, Figure 1C) and velocity [*F*(1,71) = 16.46, *p* < 0.05] (Table 2) than saline-administered controls. Following forced swim, diestrous mice travelled a significantly greater distance than their proestrous counterparts [*F*(1,71) = 17.83, *p* < 0.05] (see #, Figure 1C). There was an interaction wherein Tat exposure potentiated oxycodone’s psychomotor effects, irrespective of estrous cycle phase [*F*(1,71) = 7.40, *p* < 0.05]. Oxycodone-administered Tat(+) mice travelled a significantly greater distance than did any other group (*p* < 0.0001–0.0001; see §, Figure 1C). Expression of Tat also influenced rearing time such that Tat(+) mice significantly spent more time rearing than their respective Tat(−) controls [*F*(1,69) = 7.50, *p* < 0.05] (see *, Figure 1C). When assessed in the light-dark transition test, significant differences in anxiety-like behavior were not observed (Table 2). However, oxycodone administration interacted with Tat expression to influence motor/exploratory behavior [*F*(1,70) = 5.15, *p* < 0.05] such that oxycodone-administered Tat(+) mice made more transitions than Tat(−) oxycodone- and Tat(+) saline-administered mice (*p* = 0.0200–0.0313; see §, Table 2). Estrous cycle also interacted with Tat expression [*F*(1,70) = 8.52, *p* < 0.05] such that Tat(−) mice in the proestrous phase made significantly fewer transitions than did Tat(+) mice in the proestrous phase or Tat(−) mice in the diestrous phase (*p* < 0.0079–0.0407; Table 2).

In summary, Tat expression potentiated oxycodone-mediated psychomotor behavior. Stress enhanced this effect among diestrous, compared to proestrous, mice implicating factors associated with the HPG axis.

### 3.2. Gonadal Steroids Are Necessary for Tat to Potentiate Oxycodone-Mediated Psychostimulation

In order to determine the importance of the HPA and HPG axes in Tat-potentiated psychomotor and/or anxiety-like behavior, HPA axis receptor sites were pharmacologically blocked and circulating gonadal hormones were surgically attenuated. To achieve this, some mice were administered vehicle, antalarmin (CRF-R blocker), or RU-486 (GR blocker) concurrent with Tat induction for seven days. Some mice were OVX to remove the primary source of circulating gonadal hormones. Gonadally-intact mice were tested when in proestrus. All mice received an acute injection of saline or oxycodone (3 mg/kg) 15 min prior to behavior testing (Figure 1A).

Oxycodone significantly increased the distance travelled for all mice compared to saline administration [*F*(3,128) = 2.73, *p* > 0.05] (see †, Figure 1D). HIV-1 Tat exposure interacted with oxycodone administration to influence psychomotor behavior as assessed by the distance [*F*(1,128) = 14.42, *p* < 0.05] and velocity [*F*(1,128) = 14.56, *p* < 0.05] travelled in an open field (Figure 1D; Table 3). Irrespective of treatment with vehicle, antalarmin or RU-486, oxycodone-administered Tat(+) mice travelled a significantly greater distance (*p* < 0.0001; see §, Figure 1D) and speed (*p* < 0.0001; Table 3) than did Tat(−) controls or their saline administered counterparts. As well, there was an interaction for OVX to attenuate the Tat-potentiated increase in oxycodone-mediated distance [*F*(3,128) = 2.66, *p* < 0.05] and velocity [*F*(3,128) = 2.66, *p* < 0.05] travelled (see ‡, Figure 1D). OVX/Tat(+) mice administered oxycodone demonstrated a significant attenuation in the distance (*p* < 0.0023–0.0033; see ‡, Figure 1D) and speed (*p* < 0.0023–0.0033; Table 3) of travel compared to vehicle- or inhibitor-treated Tat(+) mice administered oxycodone, indicating the significant role ovarian hormones play modulating these effects.

In the light-dark transition test, either oxycodone [*F*(1,124) = 4.29, *p* < 0.05] (see †, Table 3) or Tat expression [*F*(1,124) = 16.66, *p* < 0.05] (see *, Table 3) significantly increased anxiety-like behavior by reducing the amount of time spent in the light zone. Pretreatment with RU-486 also increased anxiety-like behavior, reducing the amount of time spent in the light zone compared to antalarmin administration or OVX [*F*(3,124) = 2.70, *p* < 0.05] (see #, Table 3). Any pharmacological pretreatment or OVX significantly reduced the time spent time rearing compared to vehicle pretreatment [*F*(3,123) = 6.30, *p* < 0.05] (see #, Table 3). When transitions were assessed, oxycodone administration, estrous cycle phase and Tat expression interacted [*F*(3,126) = 2.75, *p* < 0.05] (see #, Table 3) such that antalarmin-treated Tat(+) mice made significantly fewer transitions than their respective Tat(−) control group (*p* = 0.046; Table 3). Conversely, antalarmin-treated Tat(−) mice made significantly more transitions than their respective Tat(−) control group (*p* = 0.0151; Table 3).

In summary, OVX attenuated Tat’s capacity to potentiate oxycodone-mediated psychostimulation. Neither CRF nor GR blockade influenced these effects, further supporting the influence of HPG factors in female mice.

### 3.3. Tat, Oxycodone, and Gonadal Steroids Interact to Influence Circulating Steroids

**Figure 2:**
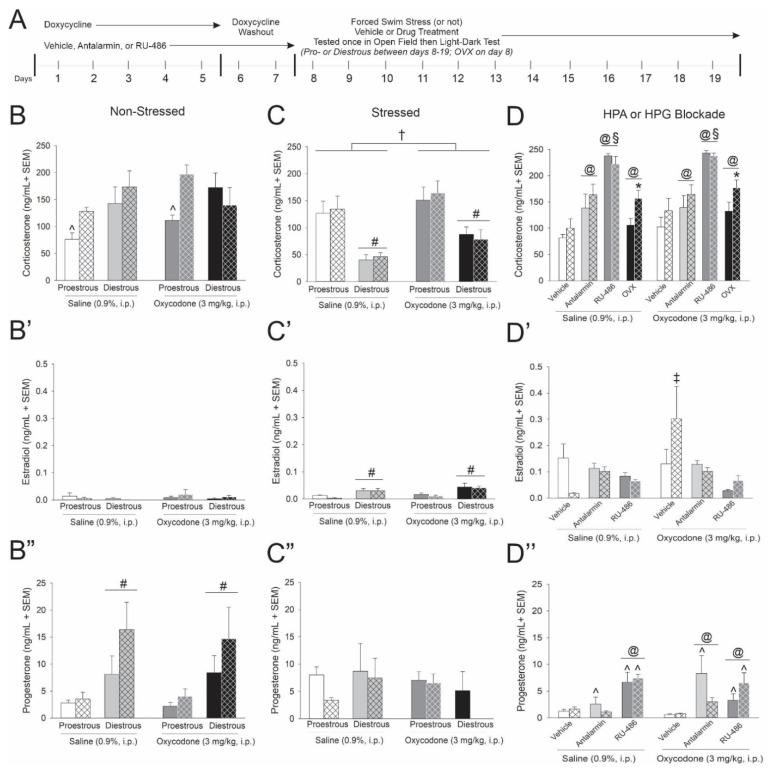
(**A**) Circulating steroid concentrations obtained from Tat-transgenic, female mice [hatched bars; Tat(+)] or control counterparts [open bars; Tat(−) controls] described in Figure 1 (*n* = 6–10/group). Corticosterone, estradiol and progesterone were assessed in (**B**–**B’’**) non-stressed, (**C**–**C’’**) stressed and (**D**–**D’’**) HPA or HPG manipulated mice. ^ indicates an interaction wherein the denoted group differs from all other groups in panelsB and D”. † indicates a main effect for oxycodone to differ from saline administered mice in panel C. § indicates an interaction wherein mice pretreated with RU-486 differ from all other groups in panel D. @ indicates an interaction wherein the denoted group differs from their respective vehicle controls in panel D and D”. * indicates a main effect of genotype wherein Tat(+) mice differ from Tat(−) controls in panel D. # indicates a main effect for proestrous mice to differ from diestrous mice in panel C, C’ and B”. ‡ indicates an interaction wherein the denoted group differs from all other groups in panel D’, *p* < 0.05.

Circulating steroid concentrations were assessed in serum ~2 h following saline or opioid injection when HPA activation is expected to be resolving. In non-stressed mice, expression of Tat interacted with estrous cycle phase to influence resolving corticosterone concentrations [*F*(1,69) = 4.60, *p* < 0.05] (see ^, Figure 2B). Irrespective of oxycodone administration, either Tat(+) or diestrous mice demonstrated significantly greater corticosterone than did proestrous Tat(−) controls (*p* = 0.0035–0.0101; Figure 2B). No significant differences were observed in circulating estradiol (Figure 2B’). Circulating progesterone was significantly greater among diestrous, compared to proestrous, mice [*F*(1,70) = 14.84, *p* < 0.05] (see #, Figure 2B’’).

Among swim stress-exposed mice, circulating corticosterone was significantly greater in oxycodone-administered mice [*F*(1,70) = 6.30, *p* < 0.05] (see †, Figure 2C) and was significantly reduced among diestrous mice [*F*(1,70) = 37.05, *p* < 0.05] (see #, Figure 2C) compared to their respective saline-administered or proestrous counterparts. Concurrently, diestrous mice had significantly greater estradiol levels than did proestrous mice [*F*(1,71) = 24.60, *p* < 0.05] (see #, Figure 2C’). No significant differences were observed in circulating progesterone among stressed mice (Figure 2C’’).

These data recapitulate the clinical endophenotype of elevated basal cortisol observed in HIV-infected individuals and further reveal HPA/HPG interactions for stress to influence circulating estradiol and progesterone.

Pharmacologically antagonizing receptors that mediate HPA feedback or removing the primary source of gonadal steroids influenced circulating corticosterone. Either pretreating mice with antalarmin or RU-486, or conducting OVX, significantly increased circulating corticosterone [*F*(3,125) = 39.65, *p* < 0.05] (see @, Figure 2D). However, only OVX Tat(+) mice demonstrated a significant corticosterone increase compared to their respective Tat(−) controls [*F*(1,125) = 6.1, *p* < 0.05] (see *, Figure 2D). Among pretreatments, GR inhibition via RU-486 increased circulating corticosterone to a greater degree than other manipulations [*F*(3,125) = 39.65, *p* < 0.05] (see §, Figure 2D). When circulating estradiol was assessed, oxycodone-administered Tat(+) mice demonstrated greater circulating concentrations than did any other group [*F*(2,97) = 3.23, *p* < 0.0001–0.0239] (see ‡, Figure 2D’). Tat exposure and HPA receptor antagonism interacted to alter circulating progesterone [*F*(2,97) = 3.43, *p* < 0.05] (see ^, Figure 2D’’). Blocking GRs via RU-486 increased circulating progesterone, irrespective of Tat exposure. However, blocking CRF-Rs via antalarmin only increased progesterone among Tat(−) control mice (*p* = 0.0002–0.0020; see ^, Figure 2D’’). Additionally, HPA receptor antagonism and oxycodone administration interacted [*F*(2,97) = 4.45, *p* < 0.05] (see @, Figure 2D’’). Blocking GRs via RU-486 increased progesterone, irrespective of oxycodone administration. Blocking CRF-Rs via antalarmin only increased progesterone among oxycodone-treated mice (*p* < 0.0001–0.0493; see @, Figure 2D’’).

In summary, OVX prompted greater circulating corticosterone among Tat(+) mice compared to Tat(−) controls, the endocrine phenotype associated with an attenuated psychomotor response to oxycodone. While CRF-R or GR blockade altered total glucocorticoid levels, no differences between Tat(−) and Tat(+) mice were observed. These data support the notion that reinstatement of HPA responsivity may involve HPG manipulation and may rescue the Tat-mediated psychomotor response to oxycodone.

### 3.4. Acute Oxycodone Interacted with Tat Exposure to Influence Hypothalamic Allopregnanolone

**Figure 3:**
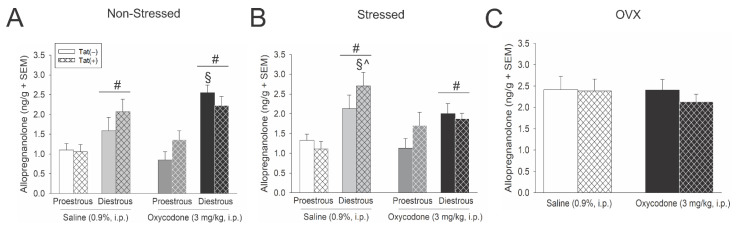
Proestrous, diestrous or ovariectomized (OVX) Tat(−) and Tat(+) mice described in Figure 1 had allopregnanolone content (ng/g) assessed in the hypothalamus in (**A**) non-stressed, (**B**) stressed, and (**C**) OVX mice (*n* = 8/group). # indicates a main effect for diestrous mice to differ from proestrous mice. § indicates an interaction wherein the denoted group differs from Tat(−) or Tat(+) proestrous mice. ^ indicates an interaction wherein the denoted group differs from oxycodone-administered, Tat(+) diestrous mice, *p* < 0.05.

Hypothalamic allopregnanolone content was greater among diestrous, compared to proestrous mice, in the non-stressed [*F*(1,56) = 36.02, *p* < 0.05] (see #, Figure 3A) and stressed paradigms [*F*(1,56) = 21.42, *p* < 0.05] (see #, Figure 3B). Moreover, Tat exposure interacted with oxycodone and estrous cycle phase to influence hypothalamic allopregnanolone content among non-stressed [*F*(1,56) = 4.02, *p* < 0.05] (see §, Figure 3A) and stressed mice [*F*(1,56) = 4.09, *p* < 0.05] (see § and ^, Figure 3B). Among non-stressed mice, oxycodone-administered Tat(−) controls demonstrated greater allopregnanolone in the diestrous phase of their cycle than did their saline-administered counterparts or any proestrous group (*p* < 0.0001–0.0066, see §, Figure 3A). Among stressed mice, Tat(+) saline-administered mice demonstrated greater allopregnanolone in the diestrous phase of their cycle than did any other proestrous group (*p* < 0.0001–0.0298, see §, Figure 3B) or their oxycodone-administered diestrous counterparts (*p* = 0.03, see ^, Figure 3B). No differences in hypothalamic allopregnanolone were observed among OVX mice (Figure 3C), despite an apparent basal increase compared to naturally-cycling mice.

In summary, hypothalamic allopregnanolone was greater among diestrous, compared to proestrous, mice and OVX apparently increased basal allopregnanolone content and obviated oxycodone- or Tat-mediated fluctuations.

## 4. Discussion

The hypotheses that the HPA and/or HPG axes contribute to Tat- and oxycodone-mediated psychomotor and affective behavior were upheld; albeit the HPA phenotype produced by Tat in females and the mechanisms contributing to these effects differed from those previously observed in males. Exposure to Tat activated the HPA stress axis, recapitulating the clinical phenotype of elevated basal cortisol seen in up to 46% of HIV^+^ patients [26,28,59,60,61]. Moreover, either Tat expression and/or oxycodone exposure, increased anxiety-like behavior in the light-dark transition test. These data recapitulate our prior findings in male Tat-tg mice [42]; however, important sex differences were also revealed. Females exposed to Tat did not demonstrate adrenal insufficiency, nor did pharmacological blockade of the HPA feedback loop attenuate Tat’s capacity to potentiate oxycodone’s psychomotor effects. One potential mechanism underlying these sex differences may involve the contribution of HPA-mediating gonadal steroid hormones. Pregnane steroids fluctuate to greater levels in females, compared to males and may help offset HPA insults. In support, we observed a full attenuation of Tat’s capacity to potentiate oxycodone’s motor effects when mice were OVX, which was accompanied by a compensatory increase in adrenal glucocorticoid. Moreover, neurosteroids such as allopregnanolone are well-characterized to play a compensatory role to curtail stress-induced HPA-activation [62,63,64]. We assessed the hypothalamic levels of allopregnanolone and observed that OVX or combined Tat and oxycodone exposure promoted an increase in endogenous hypothalamic allopregnanolone. This may serve as a central adaptive response to stress similar to our prior findings in males exposed to Tat and morphine [23]. Together, these data suggest that Tat can dysregulate glucocorticoid neuroendocrine function and that endogenous neurosteroids like allopregnanolone may be upregulated in response. These data extend findings to implicate dysregulated HPA/HPG axes in the susceptibility to neuropsychiatric complications, opioid effects, and potential abuse liability.

Sex/gender differences in HIV are understudied, contributing to some discrepancies within the extant literature. While some studies find HIV^+^ women to have an improved immunological response to cART [65,66] and slower viral progression [67] compared to HIV^+^ men, studies assessing neuroHIV outcomes reveal women to be more vulnerable to HIV-associated neurocognitive disorders [68,69,70]. However, neuroHIV vulnerability may differ based on the dimensions assessed. While women are more prone to affective disorders in the general population, large clinical studies find HIV^+^ women to have a lower prevalence of major depression or any anxiety disorder [71,72] compared to HIV^+^ men. Unfortunately, these studies are often not stratified by gender and many are not controlled by route of HIV acquisition or endocrine factors, adding heterogeneity to findings. Some studies find no gender differences in HIV-related affect [73,74]. The potential mechanisms that underlie these discrepancies in cognitive vs. affective gender differences, may involve vulnerability to stress. Domains that involve HPA axis mediation, such as generalized anxiety and depressive symptomatology, may favor women [72,75,76]. HPA axis function provides a biological basis for sex differences in stress-related psychiatric disorders [77] and may interact with HPG factors. Gonadal hormones in premenopausal women contribute to the central responsivity of the stress response [78]. In animal models, females also have a particularly robust neuroendocrine response to stress [79,80,81,82,83] and demonstrate increased hypothalamic release of CRF and downstream circulating glucocorticoids [79,83,84]. Dysregulation of HPG and HPA axes may promote neurological behavioral deficits [37,85,86]. In support, we found stressed mice to be more vulnerable to Tat/oxycodone behavioral interactions when in the diestrous phase of their cycle (higher estradiol:progesterone ratio). These data are consistent with higher estradiol levels promoting psychostimulant response to drugs of abuse in rodent models [87,88,89,90].

Psychomotor locomotion is a robust assay to quantify the response of opioids in rodents [91] and may inform abuse liability. Opioid prescriptions for HIV^+^ patients are rising [7,8,9] and opioids exert long-term capacity to promote neuroendocrine dysfunction in people [92,93]. Given the potential vulnerability that neuroendocrine dysregulation confers to substance use and affective disorders [94,95,96,97], it is important to understand the effects that HIV and opioids exert on the HPA and HPG axes. We and others find that HIV-1 Tat facilitates the rewarding and sensitizing effects of opioids [42,43,98,99]. Indeed, HPA dysregulation confers vulnerability to substance use disorders [100]. Together, these data support the notion that maintenance of neuroendocrine function may help counteract HIV-related opioid abuse liability.

There are several mechanisms that may underlie endocrine abnormalities observed in HIV patients. Hypercortisolemia is reported in a considerable proportion of HIV^+^ patients [61,101,102]. Tat may be a contributor given that the current animal model recapitulates this phenotype. There may be several mechanisms for the hypercortisolemia observed. Tat has been proposed to promote GR transcription partly via its capacity to drive positive transcription elongation factor-b accumulation on GR-responsive promoters [103]. As well, Tat (alone or in conjunction with other proinflammatory HIV proteins) may promote GR activation indirectly via cytokine activation. Elevated levels of IL-2 and IL-4 decrease the affinity of the GR for cortisol and lead to a glucocorticoid resistant state [104]. As well, the β isoform of the GR, which inhibits the active α isoform of the receptor, may be increased by proinflammatory cytokines, thereby reducing GR signaling and promoting an increase in adrenal glucocorticoid [105,106,107]. Importantly, affective neuroHIV symptoms such as depression are associated with impaired GR signaling in HIV^+^ women [108]. Another aspect of HPA dysregulation observed in HIV patients is adrenal insufficiency (observed in up to 46% patients [24,25,26,27,28,61]); however, we did not observe this in Tat-exposed females. Adrenal insufficiency based on sex as biological variable has not been well characterized in the HIV clinical population due to its multifactorial nature [24,61,109,110,111]. There may be several explanations for the lack of adrenal insufficiency in females including lower CRF-R internalization, thereby increasing sensitivity to CRF [112]. Other reasons may include lower GR receptor density and GR translocation at the level of the hypothalamus in females, thereby reducing negative feedback [113,114]. Additionally, male mice demonstrate a robust increase in hypothalamic, GR-mediated negative feedback compared to females [114], which may increase their vulnerability to HPA insults. Females may also have higher corticosterone binding globulin, reducing bioavailable corticosterone and potentially the reserve for negative HPA feedback [115]. Lastly, both circulating and central pregnane steroids fluctuate to greater levels in females compared to males and may confer protection.

The HPA axis is modulated by non-traditional-acting steroid hormones that are produced de novo in the brain (i.e., neurosteroids [62]). Neurosteroids, such as allopregnanolone, are rapidly produced in response to stress and reinstate HPA-axis homeostasis, partly via potent positive allosteric modulation of inhibitory GABA_A_ receptors [116,117,118,119]. The paraventricular nucleus of the hypothalamus (PVN) is rich in GABAergic neurons [120] and GABA_A_ receptor expression [121]. We have previously observed Tat to promote the selective loss of GABAergic interneurons within the hippocampus, particularly those that are nNOS^+^, SST^+^ or PV^+^ [122]. Increased AlloP content in hypothalamus may compensate for inhibitory deficits promoted by the loss of GABAergic interneurons. AlloP’s capacity to prevent upregulation of CRF mRNA in the PVN following adrenalectomy, attenuates CRF release in response to stress and inhibits the firing of PVN CRF neurons [121,123,124,125]. Moreover, downstream ACTH release and subsequent corticosterone secretion in rodents are attenuated by neurosteroids [62,123,124,126,127,128]. These effects are dynamic; while neurosteroids dampen the HPA response, a dysregulated HPA axis may alter neurosteroidogenesis [129,130,131,132]. We have found that HIV-1 Tat expression in mice increases neurosteroidogenesis of pregnenolone and 5α-reduced steroid metabolites, including allopregnanolone [23]. These demonstrations of upregulated allopregnanolone content in the brain are similar to those observed following central trauma and may indicate a central adaptive response to stress. In support, CNS allopregnanolone is increased following traumatic brain injury, spinal cord injury, ischemic stroke and in neurodegenerative disease models where it exerts a neuroprotective functions (reviewed in [133]). Herein, we find that OVX mice have an increase in basal, hypothalamic AlloP content concurrent with a reduction of oxycodone’s psychomotor effects, supporting its role to maintain homeostasis. Beyond its capacity to modulate GABA_A_ receptors [134], allopregnanolone is an inhibitor of L-type calcium channels [135,136] which may also attenuate Tat-mediated excitotoxicity [23,137]. Greater lability and fluctuations of pregnane steroids, such as allopregnanolone, in females may confer resilience to HPA insults such as those produced by Tat exposure.

It must be noted that Tat is not the only HIV-1 protein that may be expressed in the CNS. Other proteins including gp120 and viral protein R (VPR) may act alone or in concert with Tat to influence the HPA axis. In support, HIV-1 gp120 is observed to increase pituitary ACTH as well as circulating corticosterone [138]. Moreover, exogenous gp120 stimulates hypothalamic CRH and arginine vasopressin secretion in rats [139]. HIV-1 VPR promotes glucocorticoid hypersensitivity and was found to bind GRs directly and potentiate their signaling, in part by acting as a GR co-factor [140,141,142]. Effects of VPR to promote GR transcription may coincide with Tat’s potential capacity to co-activate GRs [61]. Future studies should assess the endocrine interactions between these proteins.

## 5. Conclusions

Together, these data demonstrate the capacity for HIV-1 Tat to dysregulate the HPA stress axis and promote vulnerability to the psychostimulant effects of oxycodone. The HPG axis likely plays an important regulatory role in these effects. Tat and oxycodone exposure altered steroid hormone production in circulation and neurosteroid formation in the hypothalamus. These effects occurred concurrent with increased psychomotor and anxiety-like behavior. Thus, maintenance of the neuroendocrine axes may exert beneficial effects on combined HIV-1 and oxycodone mediated pathology.

## Figures and Tables

**Table 1:** Motor and anxiety-like measures for Tat(−) and Tat(+) female mice assessed in open field and light-dark transition tests. Proestrus or diestrous mice were administered saline or oxycodone prior to behavioral assessment. * indicates a main effect of genotype wherein Tat(+) mice differ from Tat(−) controls. † indicates a main effect of drug condition wherein oxycodone-administered mice differs from saline-administered controls. # indicates a main effect of estrous cycle wherein diestrous mice differ from proestrous mice. § indicates an interaction wherein oxycodone-administered Tat(+) mice differ from Tat(−) controls and saline-administered controls. ^ indicates an interaction wherein saline-administered, diestrous Tat(+) mice differ from all other groups except for their respective proestrous counterparts, *p* < 0.05.

	Non-Stressed							
	Saline (0.9%, i.p.)				Oxycodone (3 mg/kg, i.p.)			
	Proestrous		Diestrous		Proestrous		Diestrous	
	Tat(−)	Tat(+)	Tat(−)	Tat(+)	Tat(−)	Tat(+)	Tat(−)	Tat(+)
**Light Zone Time (s)**	78 ± 14	88 ± 34	24 ± 5	141 ± 42 ^	61 ± 19	49 ± 13	71 ± 13	20 ± 4
**Mean Velocity (m/s)**	0.036 ± 0.003	0.030 ± 0.004	0.024 ± 0.004	0.032 ± 0.003	0.072 ± 0.005 ^†^	0.103 ± 0.017 ^§,†^	0.062 ± 0.011 ^†^	0.104 ± 0.013 ^§,†^
**Number of transitions**	15 ± 2	8 ± 1 *	6 ± 1	5 ± 1 *	19 ± 4 ^†^	15 ± 3 ^†,^*	19 ± 5 ^†^	14 ± 3 ^†,^*
**Rearing Time (s)**	19 ± 4	20 ± 3	27 ± 6 ^#^	26 ± 7 ^#^	5 ± 1	15 ± 4	60 ± 23 ^#^	33 ± 20 ^#^

**Table 2:** Motor and anxiety-like measures for Tat(−) and Tat(+) female mice assessed in open field and light-dark transition tests. Proestrus or diestrous mice were exposed to a forced swim stress prior to administration of saline or oxycodone. * indicates a main effect of genotype wherein Tat(+) mice differ from Tat(−) controls. # indicates a main effect of estrous cycle wherein diestrous mice differ from proestrous mice. § indicates an interaction wherein oxycodone-administered Tat(+) mice differ from respective Tat(−) controls and saline-administered controls. ‡ indicates an interaction wherein proestrous Tat(−) mice differ from respective Tat(+) and diestrous Tat(−) controls, *p* < 0.05.

	Forced Swim-Stressed							
	Saline (0.9%, i.p.)				Oxycodone (3 mg/kg, i.p.)			
	Proestrous		Diestrous		Proestrous		Diestrous	
	Tat(−)	Tat(+)	Tat(−)	Tat(+)	Tat(−)	Tat(+)	Tat(−)	Tat(+)
**Light Zone Time (s)**	60 ± 16	43 ± 4	112 ± 30	81 ± 28	79 ± 36	38 ± 9	45 ± 6	44 ± 10
**Mean Velocity (m/sec)**	0.002 ± 0.001	0.007 ± 0.003	0.013 ± 0.003 ^#^	0.015 ± 0.002 ^#^	0.006 ± 0.002	0.021 ± 0.008 ^§^	0.018 ± 0.003 ^#^	0.047 ± 0.009 ^#,§^
**Number of transitions**	10 ± 2	12 ± 2 ^‡^	14 ± 3 ^‡^	7 ± 1	5 ± 1	19 ± 5 ^‡,§^	14 ± 2 ^‡^	14 ± 3 ^§^
**Rearing Time (s)**	0.35 ± 0.15	1.97 ± 0.82 *	1.56 ± 0.44	2.10 ± 0.66 *	0.01 ± 0.01	1.65 ± 1.11 *	0.21 ± 0.13	1.02 ± 0.50 *

**Table 3:** Motor and anxiety-like measures for Tat(−) and Tat(+) female mice assessed in open field and light-dark transition tests. Proestrus mice were pretreated with vehicle, antalarmin, RU-486 or were ovariectomized (OVX) prior to administration of saline or oxycodone. * indicates a main effect of genotype wherein Tat(+) mice differ from Tat(−) controls. † indicates a main effect of drug condition wherein oxycodone-administered mice differ from saline-administered controls. # indicates a main effect for any HPA or HPG manipulation to differ from vehicle-pretreated mice. ^ indicates a main effect for RU-486 to differ from antalarmin or OVX. § indicates an interaction wherein oxycodone-administered Tat(+) mice differ from respective Tat(−) and saline-administered controls. @ indicates an interaction wherein antalarmin treated Tat(−) mice differ from respective Tat(+) mice and vehicle-treated Tat(−) controls. ‡ indicates an interaction wherein oxycodone-administered OVX mice differ from oxycodone-administered mice pretreated with vehicle, RU-486 and Antalarmin, *p* < 0.05.

	HPA or HPG Blockade							
	Vehicle				Antalarmin			
	Saline		Oxycodone		Saline		Oxycodone	
	Tat(−)	Tat(+)	Tat(−)	Tat(+)	Tat(−)	Tat(+)	Tat(−)	Tat(+)
**Light Zone Time (s)**	112 ± 38	95 ± 43 *	119 ± 35 ^†^	23 ± 7 ^†^	165 ± 34	100 ± 35 *	85 ± 31 ^†^	31 ± 12 ^†^
**Mean Velocity (m/sec)**	0.022 ± 0.003	0.025 ± 0.004	0.046 ± 0.009 ^†^	0.103 ± 0.009 ^†,§^	0.024 ± 0.004	0.023 ± 0.005	0.054 ± 0.013 ^†^	0.098 ± 0.015 ^†,§^
**Number of transitions**	3.4 ± 0.3	7.5 ± 1.5	14.3 ± 2.9	7.7 ± 2.3	11.8 ± 2.3 @	5.1 ± 1.1	11.2 ± 3.3	12.5 ± 4.7
**Rearing Time (s)**	62 ± 33	15 ± 4	61 ± 37	34 ± 17	9 ± 3 ^#^	12 ± 2 ^#^	4 ± 2 ^#^	7 ± 3 ^#^
	**HPA or HPG Blockade**							
	**RU-486**				**OVX**			
	**Saline**		**Oxycodone**		**Saline**		**Oxycodone**	
	**Tat(−)**	**Tat(+)**	**Tat(−)**	**Tat(+)**	**Tat(−)**	**Tat(+)**	**Tat(−)**	**Tat(+)**
**Light Zone Time (s)**	50 ± 12 ^#,^^	29 ± 8 *^,^^	83 ± 28 ^†,^^	29 ± 7 ^†,^^	129 ± 39	88 ± 30 *	137 ± 39 ^†^	27 ± 6 ^†^
**Mean Velocity (m/sec)**	0.022 ± 0.003	0.029 ± 0.004	0.061 ± 0.008 ^†^	0.093 ± 0.008 ^†,§^	0.024 ± 0.003	0.024 ± 0.002	0.045 ± 0.013 ^†,‡^	0.049 ± 0.011 ^†,§,‡^
**Number of transitions**	4.9 ± 0.6	4.3 ± 0.6	9.3 ± 2.6	10.5 ± 1.5	9.4 ± 2.5	6.8 ± 1.0	9.1 ± 2.5	10.8 ± 2.3
**Rearing Time (s)**	9 ± 2 ^#^	17 ± 4 ^#^	3 ± 1 ^#^	11 ± 2 ^#^	11 ± 2 ^#^	15 ± 2 ^#^	16 ± 11 ^#^	2 ± 1 ^#^

## Data Availability

Data available upon request.

## Supplementary Materials

The following are available online at https://www.mdpi.com/article/10.3390/v13050813/s1, Figure S1: Fold changes of tat mRNA expression using Quantitative Real-Time Polymerase Chain Reaction (qRT-PCR), Figure S2: Motor behavioral measures acquired in open field, Table S1: Primers used for qRT-PCR, Table S2: Summary of findings for primary dependent measures.
