# Supplementary material for: HIV-1 Tat Protein Promotes Neuroendocrine Dysfunction Concurrent with the Potentiation of Oxycodone’s Psychomotor Effects in Female Mice
Source: Viruses. 2021 Apr 30;13(5):813. doi: 10.3390/v13050813 (PMC8147167; doi:10.3390/v13050813)
Supplement: Supplementary file 1 [file viruses-13-00813-s001.zip › viruses-1144668-supplementary.pdf]

viruses

Supplementary Materials: HIV-1 Tat Protein Promotes Neuroendocrine Dysfunction Concurrent with the Potentiation of Oxycodone's Psychomotor Effects in Female Mice

Supplemental Methods

Quantitative Real-Time Polymerase Chain Reaction (qRT-PCR)

Primers were used for the following targets. See below for the forward and reverse primer sequences used.

Table S1. Primers used for Quantitative Real-Time PCR.

Protein	Forward Primer (5′–3′)	Reverse Primer (5′–3′)
Tat	GCCCTGGAAGCATCCAGGAAGTC	CGTCGCTGTCTCCGCTTCTTCCT
GAPDH	GGAAGCTCACTGGCATGGC	TAGACGGCAGGTCAGGTCCA

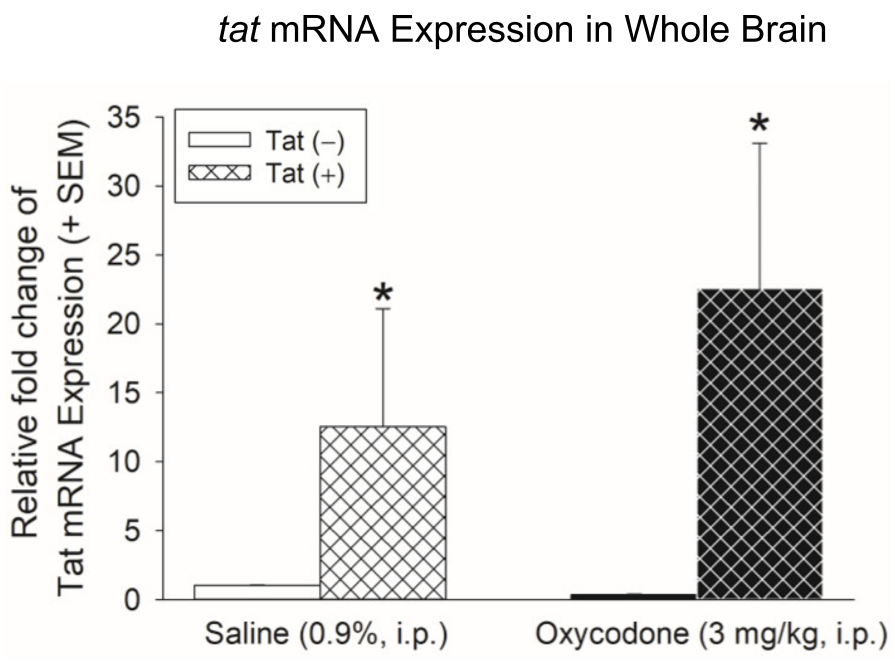

Figure S1. Fold changes of *tat* mRNA expression in the whole brains of Tat(–) (open bars) and Tat(+) (hatched bars) mice (n = 3/group) administered acute saline or oxycodone via qRT-PCR. * indicates a main effect of genotype wherein Tat(+) mice differ from Tat(–) controls, $p < 0.05$.

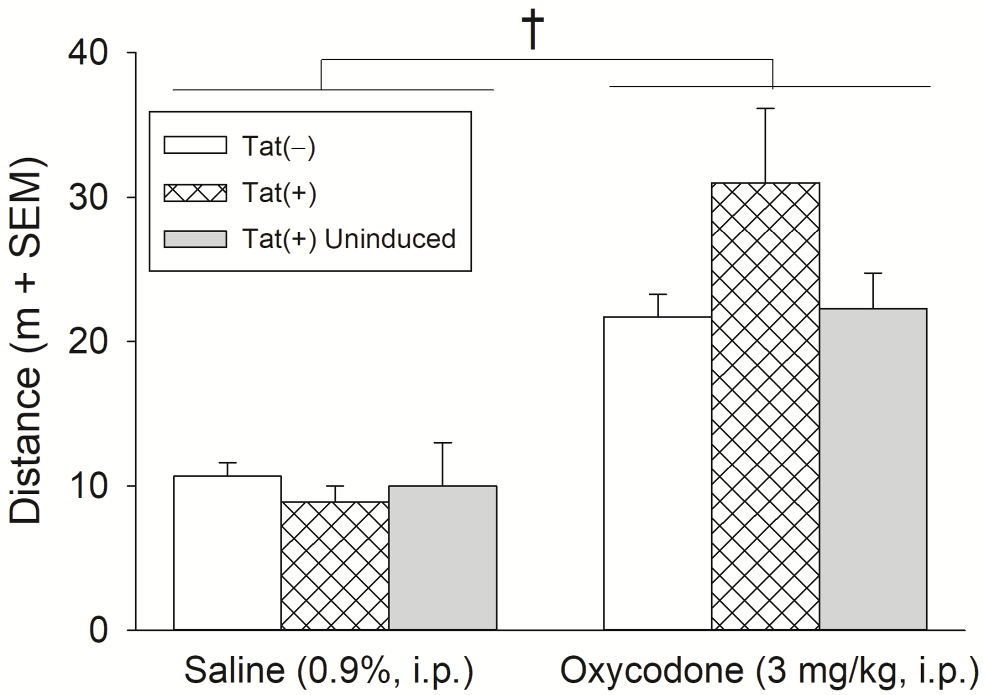

Figure S2. HIV-1 Tat-transgenic, proestrous female mice [hatched bars; Tat(+)] or control counterparts [open bars; Tat(−)] from Figure 1B of the main manuscript had transgene expression induced via doxycycline (administered once daily for 5 days with 2 days of washout; *n* = 8–10 / group). As an additional control, uninduced Tat(+) mice (grey bars; *n* = 5–6 / group) were administered saline (administered once daily 5 days with 2 days of washout) and assessed in an open field 15 minutes after administration of saline or oxycodone. Uninduced controls demonstrate that the capacity for Tat to potentiate oxycodone's psychomotor effects is not due to transgene leak in young adult Tat(+) mice. † indicates a main effect for oxycodone-administered mice to differ from saline-administered controls, $p < 0.05$.

Table S2. Summary of findings for primary dependent measures.

Behavior/Circulating Steroid	Paradigm	Outcome
Distance	Non-Stressed	Irrespective of estrous cycle phase, Tat expression potentiated oxycodone-mediated psychomotor behavior.
	Stressed	Influence of estrous cycle phase became apparent 2 h post-stress, such that mice in the diestrous phase traveled greater distances than their proestrous counterparts. Tat expression also potentiated oxycodone-mediated psychomotor behavior.
	HPA/HPG Blockade	OVX attenuated Tat-potentiated increases in oxycodone-mediated distance traveled
Light Zone Time	Non-Stressed	Tat and oxycodone increased anxiety-like behavior of diestrous to the greatest degree
	Stressed	Effects observed in non-stressed mice were obviated 2 h after forced swim stress
	HPA/HPG Blockade	Pharmacological blockade of GRs increased anxiety-like behavior compared to blockade of CRF-Rs or OVX. Tat or oxycodone exposure increased anxiety-like behavior.
Corticosterone	Non-Stressed	Exposure to Tat, or being in the diestrous phase, increased corticosterone compared to proestrous controls.
	Stressed	Oxycodone exposure increased, and being in diestrus decreased, corticosterone 2 h following stress

	HPA/HPG Blockade	Pharmacological blockade of GRs or CRF-Rs or OVX increased circulating corticosterone.<br>OVX produced increased corticosterone with Tat exposure which was not seen in other groups.
Estradiol	Non-Stressed	No significant effect was found
	Stressed	Diestrous mice had significantly greater estradiol levels than did proestrous mice 2 h post-stress
	HPA/HPG Blockade	Tat and oxycodone exposure increased estradiol and this effect was obviated by GR or CRF-R blockade
Progesterone	Non-Stressed	Circulating progesterone was significantly greater among diestrous, compared to proestrous, mice
	Stressed	No significant effect was found
	HPA/HPG Blockade	Blocking GRs increased circulating progesterone, irrespective of Tat exposure; however, blocking CRF-Rs only increased progesterone among Tat(−) control mice
